# Comparative Effects of Di(*n*-Butyl) Phthalate Exposure on Fetal Germ Cell Development in the Rat and in Human Fetal Testis Xenografts

**DOI:** 10.1289/ehp.1408248

**Published:** 2014-12-16

**Authors:** Sander van den Driesche, Chris McKinnell, Ana Calarrão, Laura Kennedy, Gary R. Hutchison, Lenka Hrabalkova, Matthew S. Jobling, Sheila Macpherson, Richard A. Anderson, Richard M. Sharpe, Rod T. Mitchell

**Affiliations:** MRC Centre for Reproductive Health, The Queen’s Medical Research Institute, The University of Edinburgh, Edinburgh, United Kingdom

## Abstract

**Background:**

Phthalate exposure induces germ cell effects in the fetal rat testis. Although experimental models have shown that the human fetal testis is insensitive to the steroidogenic effects of phthalates, the effects on germ cells have been less explored.

**Objectives:**

We sought to identify the effects of phthalate exposure on human fetal germ cells in a dynamic model and to establish whether the rat is an appropriate model for investigating such effects.

**Methods:**

We used immunohistochemistry, immunofluorescence, and quantitative real-time polymerase chain reaction to examine Sertoli and germ cell markers on rat testes and human fetal testis xenografts after exposure to vehicle or di(*n*-butyl) phthalate (DBP). Our study included analysis of germ cell differentiation markers, proliferation markers, and cell adhesion proteins.

**Results:**

In both rat and human fetal testes, DBP exposure induced similar germ cell effects, namely, germ cell loss (predominantly undifferentiated), induction of multinucleated gonocytes (MNGs), and aggregation of differentiated germ cells, although the latter occurred rarely in the human testes. The mechanism for germ cell aggregation and MNG induction appears to be loss of Sertoli cell–germ cell membrane adhesion, probably due to Sertoli cell microfilament redistribution.

**Conclusions:**

Our findings provide the first comparison of DBP effects on germ cell number, differentiation, and aggregation in human testis xenografts and *in vivo* in rats. We observed comparable effects on germ cells in both species, but the effects in the human were muted compared with those in the rat. Nevertheless, phthalate effects on germ cells have potential implications for the next generation, which merits further study. Our results indicate that the rat is a human-relevant model in which to explore the mechanisms for germ cell effects.

**Citation:**

van den Driesche S, McKinnell C, Calarrão A, Kennedy L, Hutchison GR, Hrabalkova L, Jobling MS, Macpherson S, Anderson RA, Sharpe RM, Mitchell RT. 2015. Comparative effects of di(*n*-butyl) phthalate exposure on fetal germ cell development in the rat and in human fetal testis xenografts. Environ Health Perspect 123:223–230; http://dx.doi.org/10.1289/ehp.1408248

## Introduction

*In utero* exposure of rats to high doses of certain phthalate esters, such as diethylhexyl phthalate (DEHP) or di(*n*-butyl) phthalate (DBP), impairs steroidogenesis by the fetal testis, resulting in postnatal disorders such as hypospadias, cryptorchidism, and impaired spermatogenesis (Drake et al. 2009; Johnson et al. 2012; Lehmann et al. 2004; Thompson et al. 2004). In humans, these disorders are thought to comprise a “testicular dysgenesis syndrome” (TDS) (Skakkebaek et al. 2001), for which the DBP-exposed Wistar rat may be a useful model to dissect the underlying mechanisms (Fisher et al. 2003; Sharpe and Skakkebaek 2008). However, in contrast to the inhibitory effects of DBP exposure on the fetal rat testis, the human fetal testis appears to be insensitive to the steroidogenic effects of DBP based on studies involving *in vitro* and xenograft models (Albert and Jégou 2014; Heger et al. 2012; Lambrot et al. 2009; Mitchell et al. 2012; Spade et al. 2014).

DEHP/DBP exposure also induces germ cell effects in the fetal rat testis, namely, induction of multinucleated gonocytes (MNGs) (Ferrara et al. 2006; Mylchreest et al. 2002; Parks et al. 2000) and aggregation of germ cells in the seminiferous cords (Barlow and Foster 2003; Kleymenova et al. 2005). These changes are evident only from embryonic day (E) 19.5 to E21.5 in the rat, and are thus confined to differentiated germ cells [i.e., no octamer-binding transcription factor 3/4 (OCT3/4) expression) (Ferrara et al. 2006; Jobling et al. 2011). Indirect evidence (Jobling et al. 2011; Kleymenova et al. 2005) suggests that these germ cell changes may be secondary to effects on Sertoli cells. However, DEHP/DBP exposure also induces a reduction in germ cell number that is divorced temporally from aggregation. This effect is confined to the period in the rat when germ cells are undifferentiated (expressing OCT3/4) and proliferating, namely, E13.5–E17.5 (Jobling et al. 2011), and can cause up to 40% reduction in germ cell number by birth (Jobling et al. 2011).

DEHP/MEHP induces germ cell loss and MNGs *in vitro* using human fetal testis explants (Chauvigné et al. 2009; Habert et al. 2009; Lambrot et al. 2009; Lehraiki et al. 2009; Muczynski et al. 2012), and DBP exposure induces MNGs in human fetal testis xenografts (Heger et al. 2012). However, none of these studies determined whether the phthalate effects were dependent on the stage of germ cell differentiation, which appears to be critical *in vivo* in rats.

In the present study we sought to *a*) compare the effects of DBP exposure on germ cell aggregation and MNG induction in the rat and in an established human fetal testis xenograft model (Mitchell et al. 2010, 2012); *b*) determine whether these changes result from impaired Sertoli–germ cell interaction at the membrane level; *c*) establish whether DBP-induced germ cell loss occurs in the human fetal testis xenograft model; and *d*) determine whether effects are restricted to the undifferentiated germ cell population. By answering these questions, we could identify the potential effects of phthalate exposure on fetal human germ cells and establish that the rat may be an appropriate model for investigating underlying mechanisms (and their consequences). This is important, considering the disparity in phthalate effects on fetal testis steroidogenesis in the rat and human and that fetal germ cells ultimately give rise to the next generation. Fetal germ cells are also believed to be the cell of origin for testicular germ cell cancers in young men (Rajpert-De Meyts 2006).

## Materials and Methods

*Ethics statement*. We treated animals humanely and with regard for alleviation of suffering, according to the Animal (Scientific Procedures) Act 1986 (http://www.legislation.gov.uk/ukpga/1986/14/contents) and approval by the UK Home Office. Studies were conducted under Project Licence (PPL 60/4564) following review by the University of Edinburgh Animal Research Ethics Committee. We obtained human fetal testes from women undergoing elective termination of pregnancy, who gave written consent; ethical approval was obtained from the Lothian Research Ethics Committee (LREC-08/S1101/1).

*DBP treatment of rats*. Wistar rats (Harlan, Derby, UK) were housed in ventilated cages (2000P cages; Tecniplast, Buguggiate, Italy) with 4–6 adult female rats per cage, and had *ad libitum* access to sterile water and a soy-free breeding diet [RM3(E); SDS, Dundee, Scotland]. We carefully controlled housing conditions: lights on at 0700 and off at 1900 hours; temperature, 19–21°C; humidity, 45–65%; GOLD shavings and LITASPEN standard bedding (SPPS, Argenteuil, France). Animals were housed for a minimum of 2 weeks prior to use in experimental studies. We randomly allocated time-mated females to receive either 0 (control), 4, 20, 100, or 500 mg/kg DBP (99% pure; Sigma-Aldrich, Dorset, UK) in 1 mL/kg corn oil daily by oral gavage. Treatments were administered between 0900 and 1030 hours, commencing on E13.5 until the day prior to culling (or as indicated otherwise). All treatments were performed in a single animal facility at the University of Edinburgh. The weight of the female rats prior to the start of treatment was 266.4–319.8 g, and we observed no generalized adverse effects of the exposure to DBP. There was no significant effect of the treatment on litter size or sex ratio. We sampled male offspring on E17.5, E21.5, or postnatal day (PND) 4—time points chosen to reflect the period before, during, and after the appearance of DBP-induced MNGs and gonocyte aggregation. We used 12–14 animals from three to five litters per exposure group, and all experiments reported included animals from each of these litters. Pregnant dams were killed by CO_2_ inhalation followed by cervical dislocation. Fetuses were removed, decapitated, and placed in ice-cold phosphate-buffered saline (PBS; Sigma-Aldrich). PND4 pups were housed with their natural mothers from birth and were killed by cervical dislocation. Fetuses and pups were transported immediately to the laboratory, and testes were removed by microdissection. Embryonic testes were fixed for 1 hr in Bouin’s fixative (3 hr for PND4 testes) then transferred to 70% ethanol. We used representative fetuses for the quantitative and immunohistochemical experiments. For each E21.5 fetus, one testis was fixed as described above, and the other testis was snap frozen and stored at –70°C for gene expression analysis.

*Human fetal testis xenografts*. Male CD1 nude mice (Charles River, Margate, UK) were used as host mice for human fetal testis xenografts. Mice were housed in individually ventilated cages with free access to sterile food and water. Human fetal testis tissue (collected at 14–20 weeks; *n* = 7) was xenografted subcutaneously into host mice as previously described (Mitchell et al. 2010, 2012). Host mice carrying human fetal testes xenografts (5–6 per mouse) were subcutaneously injected every 72 hr (three times per week) with human chorionic gonadotropin (hCG) (20 IU, Pregnyl; Organon Laboratories, Cambridge, UK) in 0.9% (wt/vol) saline containing 1% (vol/vol) fetal bovine serum in order to mimic the normal *in utero* environment. We then treated host mice with vehicle or 500 mg/kg/day DBP for 21 days as described previously (Mitchell et al. 2012). We fixed xenografts in Bouin’s fixative for 1 hr, transferred them to 70% ethanol, and processed them into paraffin blocks using standard procedures.

*Immunohistochemistry*. We used immunohistochemistry for VASA [DEAD (Asp-Glu-Ala-Asp) box polypeptide 4/mouse VASA homologue; DDX4/MVH] to identify germ cells in rat testes for analysis of aggregation using image analysis. Similarly, we performed immunohistochemistry for MAGE-A4 (melanoma antigen family A, 4; expressed only in differentiated germ cells) and anti-Müllerian hormone (AMH; in Sertoli cells) on human fetal testis xenograft sections. Antibodies and detection reagents are listed in Table 1. For each experiment, we included a negative control (primary antibody replaced with appropriate normal serum). We used protocols described previously (Jobling et al. 2011; McKinnell et al. 2013; Mitchell et al. 2010; van den Driesche et al. 2012).

**Table 1:** Antibodies used for immunohistochemistry and immunofluorescence.

Primary antibody	Source	Dilution	Secondary antibody	Visualization
Melanoma-associated antigen 4 (MAGE-A4; IHC)	Gift^*a*^	1:20	GAM-b^*b*^	DAB^*c*^
VASA [DEAD (Asp-Glu-Ala-Asp) box polypeptide 4 (DDX4); mouse VASA homologue (MVH)]	Abcam	1:80	GAR-b^*b*^	DAB^*c*^
Anti-Müllerian hormone (AMH)	Santa Cruz Biotechnology	1:500	RAG-b^*b*^	DAB^*c*^
Adenomatous polyposis coli (APC)	Fisher Scientific	1:750	GAR-p^*b*^	Tyr-fl^*d*^
Espin	BD Transduction Labs	1:4,000	GAM-p^*b*^	Tyr-Cy3
N-Cadherin	Zymed Laboratories	1:4,000	GAM-p^*b*^	Tyr-fl^*d*^
Vimentin	DAKO	1:750	GAM-p^*b*^	Tyr-Cy3^*d*^
MAGE-A4 (IF)	Gift^*a*^	1:100	ChAM-p^*b*^	Tyr-Cy5^*d*^
Octamer-binding transcription factor 3/4 (OCT3/4)	Santa Cruz Biotechnology	1:100	ChAG-p^*b*^	Tyr-Cy3^*d*^
Ki67	DAKO	1:200	ChAM-p^*b*^	Tyr-fl^*d*^
Abbreviations: ChAG-p, chicken anti-goat peroxidase; ChAM-p, chicken anti-mouse peroxidase; DAB, 3,3´-diaminobenzidine tetrahydrochloride; GAM-b, goat anti-mouse biotin; GAM-p, goat anti-mouse peroxidase; GAR-b, goat anti-rabbit biotin; GAR-p, goat anti-rabbit peroxidase; IHC, immunohistochemistry; IF, immunofluorescence; MAGE-4, melanoma antigen family A, 4; RAG-b, rabbit anti-goat biotin; Tyr-Cy3, tyramide Cy3; Tyr-Cy5, tyramide Cy5; Tyr-fl, tyramide fluorescein. ^***a***^Gift from G. Spagnoli, University Hospital, Basel, Switzerland. ^***b***^DAKO (Ely, Cambridgeshire, UK). ^***c***^Vector Labs (Peterborough, UK). ^***d***^PerkinElmer (Billerica, MA, USA).				

*Double immunofluorescence for adenomatous polyposis coli (APC)/espin, vimentin/N-cadherin, and APC/N-cadherin*. To determine whether germ cell aggregation in rats resulted from impaired Sertoli–germ cell interaction at the cell membrane, we performed double fluorescence immunohistochemistry for N-cadherin plus vimentin and for APC plus espin; results were analyzed using confocal laser scanning microscopy. We costained vehicle- and DBP-exposed human fetal testis xenograft sections for N-cadherin and APC using previously described procedures (Jobling et al. 2011; McKinnell et al. 2013). Antibodies and detection reagents are listed in Table 1.

*Triple immunofluorescence for OCT3/4, MAGE-A4, and Ki67*. To investigate whether DBP exposure of human fetal testis xenografts affected germ cell number or proliferation, we undertook triple fluorescence immunohistochemistry for the undifferentiated (pluripotent) germ cell marker OCT3/4, the differentiated germ cell marker MAGE-A4, and the proliferation marker Ki67, as described previously (Mitchell et al. 2010). Antibodies and reagents are listed in Table 1.

**Figure 1:**
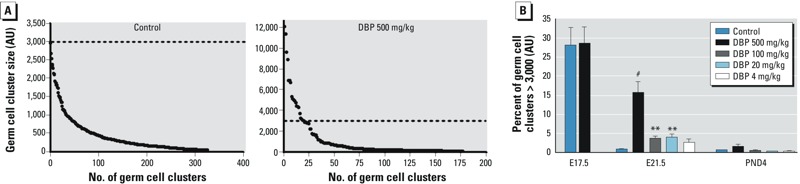
(*A*) Analysis of the number and size of germ cell aggregates for a representative control rat (left) and a DBP-exposed rat (right) at E21.5. The dashed lines indicate clusters > 3,000 AU, the arbitrary cut-off point; note the different scales used for vehicle and DBP exposure. (*B*) Dose dependence of DBP-induced germ cell aggregation at E21.5. Unless otherwise indicated, exposure took place daily on E13.5–16.5 (E17.5), E13.5–20.5 (E21.5), or E13.5–21.5 (PND4). Values shown are mean ± SE of 5 animals (representing 5 litters) per exposure group. Data were analyzed by one-way ANOVA followed by Bonferroni post hoc test. **p < 0.01, and ^#^*p* < 0.001, compared with the respective control.

*Measurement of germ cell aggregation in rat testes*. Because all germ cells in the perinatal rat testis immunostain intensely for VASA in the cytoplasm, we were able to use VASA immunohistochemistry to develop an objective method for measuring germ cell aggregation, based on average germ cell cluster size, in testes from control and DBP-exposed rats at E17.5, E21.5, and PND4. We used stereological methods adapted from previous studies to evaluate fetal Leydig cell aggregation (Mahood et al. 2005, 2007) using sections from at least five fetuses from three to five litters. In E21.5 controls, germ cells were in clusters that varied in size from < 20 to 2,800 arbitrary units (AU), with most sized < 1,000 AU (Figure 1A). In E21.5 DBP-exposed animals, clusters were fewer and, on average, 15% of seminiferous cords contained clusters > 3,000 AU, a size rare in controls (Figure 1A). A cutoff of > 3,000 AU discriminated best between control and DBP-exposed animals at E21.5 and was therefore used for all aggregation analyses. For determination of germ cell number at E21.5 after *in utero* exposure to DBP from E13.5–E15.5 or E19.5–E20.5, we also used VASA staining and stereology as described by Jobling et al. (2011).

*Quantification of MNGs in human fetal testis xenografts and fetal rat testes*. AMH is expressed in Sertoli cell cytoplasm but not in germ cells, so we used AMH immunostaining to facilitate identification of germ cells and MNGs, and to quantify the frequency of MNGs in human fetal testis xenografts and in E21.5 fetal rat testes. We analyzed two sections per xenograft and one complete testis cross section for fetal rats using an Axio-Imager microscope (Carl Zeiss Ltd, Cambridge, UK) fitted with a Hitachi HV-C20 camera (Hitachi Denshi Europe, Leeds, UK) and a Prior automatic stage (Prior Scientific Instruments Ltd., Cambridge, UK). We used Image-Pro 6.2 software with the Stereologer plug-in (MagWorldwide, Wokingham, UK) to select random fields within the tissue (15–25 fields per section). All germ cells and MNGs in each field were counted separately using the Stereologer manual tagging function, and the prevalence of MNGs was calculated as a percentage of the total counted (≥ 350 germ cells plus MNGs per section).

*Quantification of germ cell differentiation and proliferation in human fetal testis xenografts*. We generated high-resolution, tiled confocal scanning laser microscopy images of complete human fetal testis xenograft sections costained for OCT3/4, MAGE-A4, and Ki67 and then quantified germ cell subpopulations and proliferation indices. We counted all germ cells within each section according to their protein expression profile and proliferation status using Zen 2011 software (Carl Zeiss Ltd.). Each xenografted testis was implanted into two to four recipient mice for each treatment. We determined germ cell number and differentiation status for each xenograft; we then compiled the average germ cell number per treatment and fetus. The total number of germ cells counted ranged from 43 to 457 in different xenografts.

*Image capture*. We examined and photographed nonfluorescent images using a Provis AX70 microscope (Olympus Optical, London, UK) fitted with a Canon DS6031 digital camera (Canon Europe, Amsterdam, the Netherlands). We captured all fluorescent images using a Zeiss LSM 710 confocal microscope (Carl Zeiss Ltd.). We compiled images using Adobe Photoshop v12 (Adobe, San Jose, CA, USA).

*Gene expression analysis in E21.5 rat fetal testes samples*. For quantitative analysis of gene expression, we extracted and used total RNA from E21.5 testes to prepare random hexamer-primed cDNA (van den Driesche et al. 2012). We performed quantitative real-time polymerase chain reaction (qRT-PCR) using the ABI Prism Sequence Detection System (Applied Biosystems, Grand Island, NY, USA). We determined the expression of rat *Apc*, *Espn* (espin), *Cdh2* (cadherin 2), and *Vim* (vimentin) mRNA using the Roche Universal Probe Library (Roche Applied Sciences, Burgess Hill, UK) [*Apc* forward primer: 5´-CTTC​GTGT​ACGG​CAGC​TCTT-3´, reverse primer: 5´-GCAG​TTTC​ATGC​TTGC​TCTG-3´ (probe 127, catalog no. 04693639001); *Espn* forward primer: 5´-CACC​CTCT​CCAA​CTAT​GACT​CC-3´, reverse primer 5´-GCTC​TGTA​AGTC​TGAG​GATC​TGG-3´, probe 25, catalog no. 04686993001; *Cdh2* forward primer: 5´-TGTT​CCAG​AGGG​ATCA​AAGC-3´, reverse primer: 5´-GAGA​GGAT​CCTG​TACC​TCAG​CA-3´, probe 122, catalog no. 04693566001; *Vim* forward primer: 5´-CAGG​AAGC​TGCT​GGAA​GG-3´, reverse primer: 5´-GGAA​GTGA​CTCC​AGGT​TAGT​TTCT-3´, probe 108, catalog no. 04692276001. We corrected expression of each gene using a ribosomal 18S internal control (catalog no. 4308329; Applied Biosystems). We analyzed all samples in triplicate and made relative comparison to adult testis control cDNA using the ddCt method. We analyzed 12 animals for the control group (from three litters) and 14 animals for the 500 mg/kg/day DBP group (from five litters).

*Statistical analysis*. Most of the rat data was derived from one animal per litter and was thus analyzed using either Student’s *t*-test or one-way analysis of variance (ANOVA) followed by the Bonferroni post hoc test; for experiments in which several animals were used per litter (mRNA expression analysis), we accounted for within-litter effects using two-factor ANOVA. We analyzed human fetal testis xenograft experiments using the paired *t*-test (MNG analysis) or two-way ANOVA (germ cell counts), the latter to take into account within-fetus effects. We performed analyses using GraphPad Prism 5 (GraphPad Software Inc., La Jolla, CA, USA).

## Results

**Figure 2:**
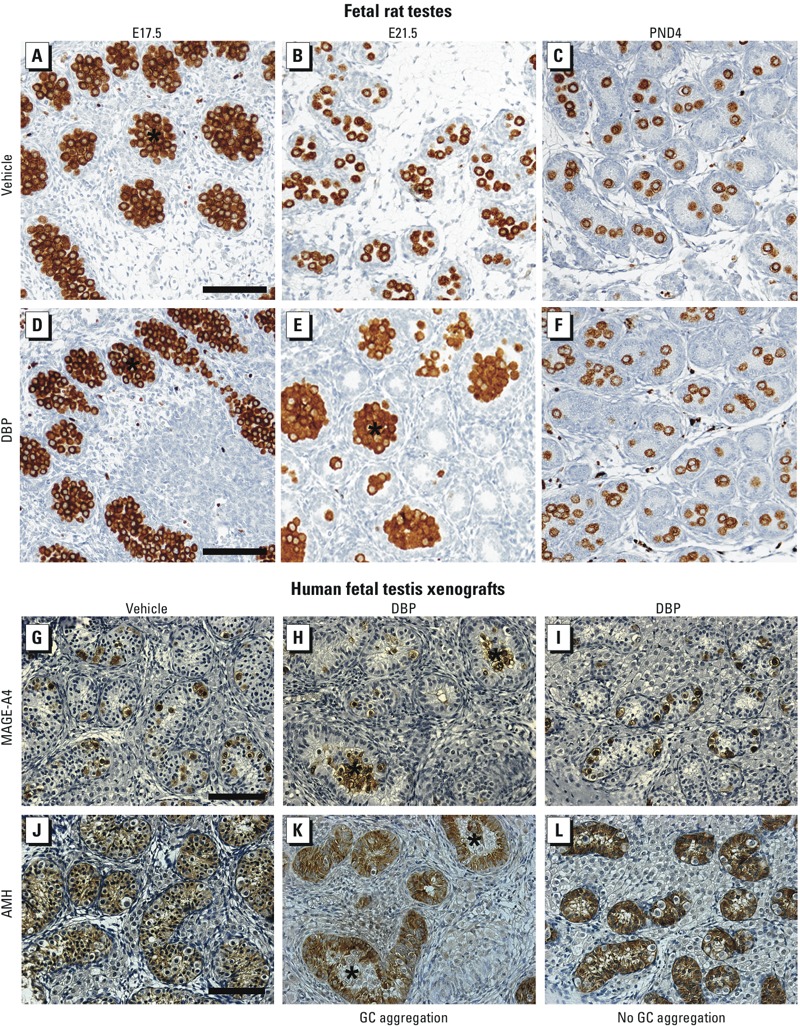
Germ cell (GC) distribution and GC aggregation in the rat at E17.5, E21.5, and PND4 after exposure to vehicle (control; *A–C*) or DBP at 500 mg/kg (*D–F*) and in human fetal testis xenografts after exposure to vehicle (*G,J*) or 500 mg/kg DBP (*H,I,K,L*). (*A–F*) Distribution of GCs visualized by immunostaining for VASA (DDX4/MVH); at E21.5, GC aggregation (asterisks) was present in testes of DBP-exposed animals (*E*), but GC distribution was normal in testes from vehicle-exposed animals (*B*). (*G–L*) Consecutive sections of human fetal testis xenografts from vehicle- and DBP-exposed hosts immunostained for MAGE-A4 (*G–I*) or AMH (*J–L*). Note that GC aggregations were found in only 4 of 54 DBP-exposed xenografts, all from one fetus. Bars = 50 μm (*A–F*) and 100 μm (*G–L*).

*Germ cell aggregation in fetal rat testes and human fetal testis xenografts*. DBP-induced rat germ cell aggregation was evident at E21.5 but largely resolved by PND4 (Figure 2A–F), findings we confirmed quantitatively (Figure 1B). Unexpectedly, E17.5 germ cells appeared to be aggregated in both control and DBP-exposed animals (Figure 2A,D). However, whereas these germ cell aggregates underwent “disaggregation” in controls between E17.5 and E21.5, this process failed to occur by E21.5 in DBP-exposed animals (Figure 2E). We evaluated the dose dependence of DBP-induced germ cell aggregation at E21.5 and found that aggregation was induced by exposure to DBP at all doses of ≥ 20 mg/kg (Figure 1B).

Using xenograft sections immunostained for MAGE-A4 (Figure 2G–I) or for OCT3/4 (not shown), we found germ cell aggregation in only 4 of 54 DBP-exposed xenografts from seven fetuses, and these 4 xenografts were all derived from a single fetus (Figure 2H). The remaining 50 DBP-exposed xenografts were indistinguishable from vehicle-exposed xenografts (Figure 2G,I). We confirmed this by immunostaining for the Sertoli cell marker AMH (Figure 2J–L). We concluded that DBP-exposure resulted only in sporadic germ cell aggregation in xenografted human testes, but when aggregation was induced, it affected differentiated [MAGE-A4–positive (MAGE-A4^pos^)] germ cells, as in rats.

**Figure 3:**
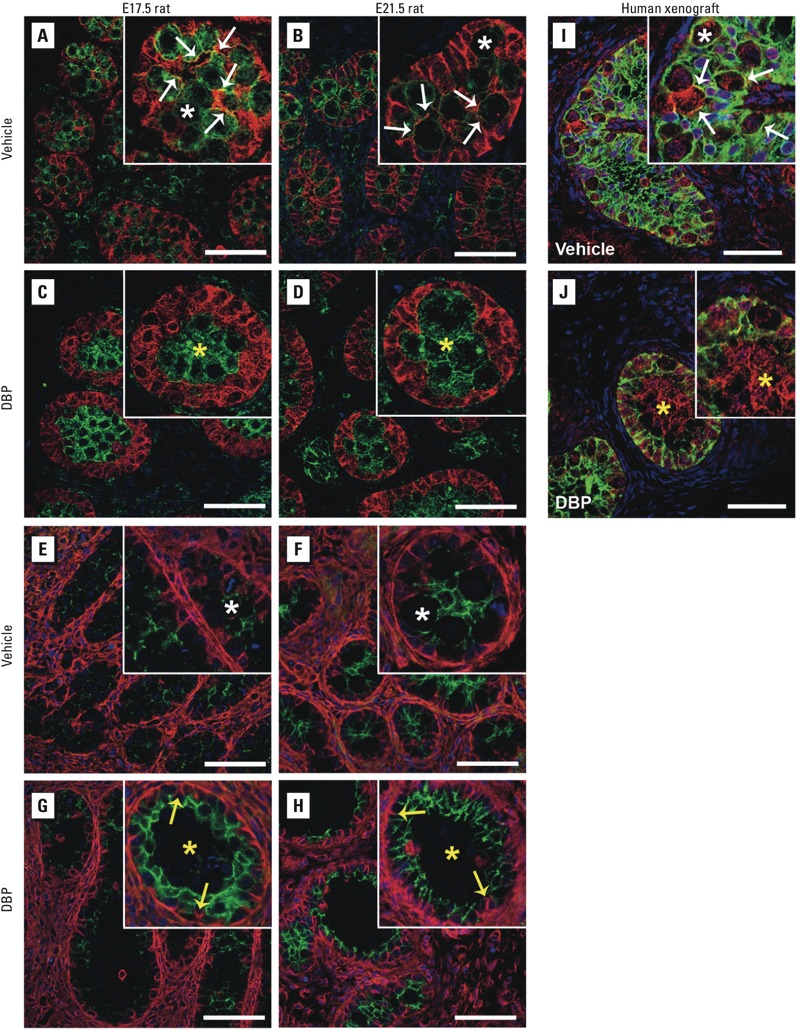
Effect of exposure to vehicle or DBP (500 mg/kg/day) on Sertoli–germ cell interactions in fetal rat testes at E17.5 and E21.5 (*A–H*) and in human fetal testis xenografts (*I,J*). Representative photomicrographs of fetal rat testes on E17.5 and E21.5 show immunoexpression of APC (green) in germ cells and espin (red) in Sertoli cells (*A–D*), and vimentin (red) and N-cadherin (green) in Sertoli cells (*E–H*). (*I,J*) APC (red) and N-cadherin (green) immunoexpression were evaluated in xenografts from hosts treated with vehicle (*I*) and DBP (*J*). Insets, higher magnification of seminiferous cords. In rats, yellow staining (APC and espin colocalization; white arrows) was absent in DBP-exposed samples at all ages (*C–D*) compared with vehicle-exposed controls (*A–B*). Similarly, vimentin and N-cadherin costaining demonstrated a lack of apical Sertoli cell cytoplasmic extensions after DBP exposure (yellow arrows) in rats, indicative of withdrawal of Sertoli cell cytoplasm from around and between the germ cells (*E–H*). Similar loss of Sertoli-germ cell membrane interaction was observed in the limited human fetal testis xenografts that exhibited germ cell aggregation after exposure to DBP (*J*). White asterisks indicate normal germ cells, and yellow asterisks indicate abnormal germ cell aggregation. In rats, treatment was administered daily from E13.5 until the day before sampling (E17.5 or E21.5). Bars = 50 μm.

**Figure 4:**
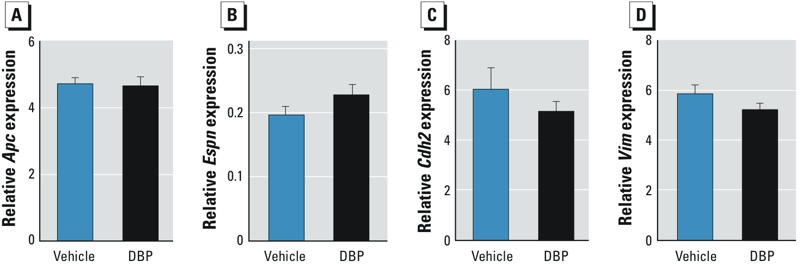
Effect of *in utero* exposure of rats to vehicle or DBP (500 mg/kg/day) on the expression of *Apc* (*A*), *Espn* (*B*), *Cdh2* (*C*), and *Vim* (*D*) in testes at E21.5 as evaluated by qRT-PCR. Values shown are mean ± SE representing 12 animals in the vehicle group (from three litters) and 14 animals in the DBP group (from five litters).

*Sertoli–germ cell interaction*. To study the role of Sertoli–germ cell interactions in DBP-induced germ cell aggregation, we examined expression of cell–cell adhesion molecules, APC, espin, vimentin, and N-cadherin (Bartles et al. 1996; Chen et al. 1999; Ivaska et al. 2007; Kleymenova et al. 2005; Strelkov et al. 2003; Tanwar et al. 2011; Vogl et al. 2008). In control rats at E17.5 and E21.5, espin was expressed in distal Sertoli cell cytoplasm that extended into “fingers” that encircled each germ cell (Figure 3A,B). APC expression was restricted to germ cells but colocalized with espin (expressed in Sertoli cells) where Sertoli and germ cells were apposed (visible as yellow staining in insets of Figure 3A,B); we interpreted this as evidence for Sertoli–germ cell interaction at the membrane level, and it was evident at all ages in controls. In contrast, in DBP-exposed rats at E17.5 and E21.5, Sertoli cell cytoplasm was not evident between the germ cells, and there was no overlapping of espin and APC expression (Figure 3C,D), indicative of the absence of direct Sertoli–germ cell membrane contact, coincident with germ cell aggregation. However, by PND4, Sertoli cell espin expression in testes of DBP-exposed rats was largely restored to normal, with cytoplasmic “fingers” extending around individual germ cells and interacting with APC at the membrane level, as in controls (data not shown). Immunostaining for the Sertoli cell filaments vimentin and N-cadherin showed that the former was confined to basal and the latter to apical Sertoli cell cytoplasm, but both showed evidence for retraction in DBP-exposed rats, similar to that found for espin (Figure 3E–H). Because mRNA expression for *Apc*, *Espn*, *Cdh2*, and *Vim* was unchanged at E21.5 after DBP exposure (Figure 4), we presume that the DBP-induced microfilament changes resulted from protein redistribution, although DBP-induced protein degradation and/or translational regulation cannot be excluded.

Immunostaining for APC and N-cadherin in control human fetal testis xenografts demonstrated Sertoli cell cytoplasm distribution around the germ cells (Figure 3I,J), similar to that in rats. However in the few seminiferous cords that exhibited germ cell aggregation after exposure to DBP, there was a lack of Sertoli cell cytoplasmic “fingers” extending around the germ cells, again comparable to the findings in rats.

**Figure 5:**
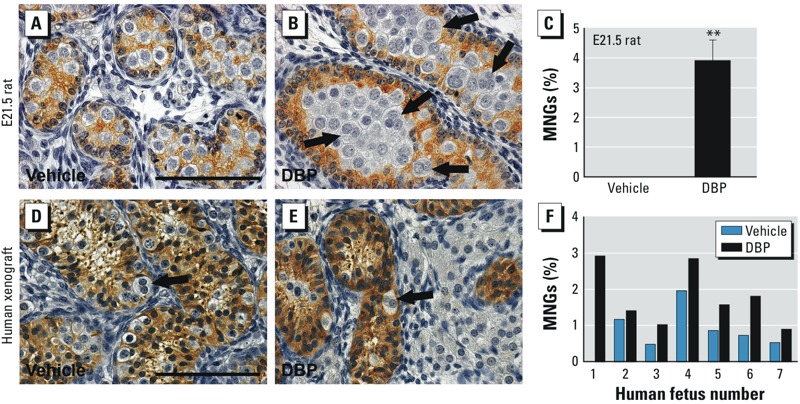
Effect of exposure to vehicle or DBP (500 mg/kg/day) on the induction of multinucleated gonocytes (MNGs) in rat testes at E21.5 (*A–C*) and in human fetal testis xenografts (*D–F*). AMH immunostaining (brown) highlights an increased number of MNGs (arrows) in both rat (*B*) and human (*E*) fetal testis samples after DBP exposure; these results were confirmed by quantification (*C,F*). MNG quantification in rat testis (*C*) represents 4 control animals and 4 DBP-exposed animals, with one section per testis analyzed. For xenografts (*F*), two sections were quantified from 1–5 grafts from seven different fetuses (numbered 1–7); *p* = 0.0034 for DBP exposure compared with vehicle. For *A–E*, bars = 100 μm. **p < 0.01 compared with the respective vehicle group.

*MNGs in E21.5 rat testes and in human fetal testis xenografts after exposure to DBP*. We used AMH-stained sections in which germ cells remained unstained to determine the appearance of MNGs in fetal rat testes and human fetal testis xenografts after DBP exposure. In E21.5 testes from DBP-exposed rats, we observed a significant increase in MNGs (expressed as a percentage of all germ cells) compared with the vehicle-exposed group (controls 0%; DBP 3.9%, *p* = 0.0016; Figure 5A–C). In human fetal testis xenografts, we observed a low frequency (0.8%) of MNGs in vehicle-exposed controls, similar to that described by Heger et al. (2012). We also observed a significant increase in incidence (1.76%) of MNGs in xenografts from DBP-exposed animals compared with vehicle-exposed animals (*p* = 0.0034; Figure 5D–F).

**Figure 6:**
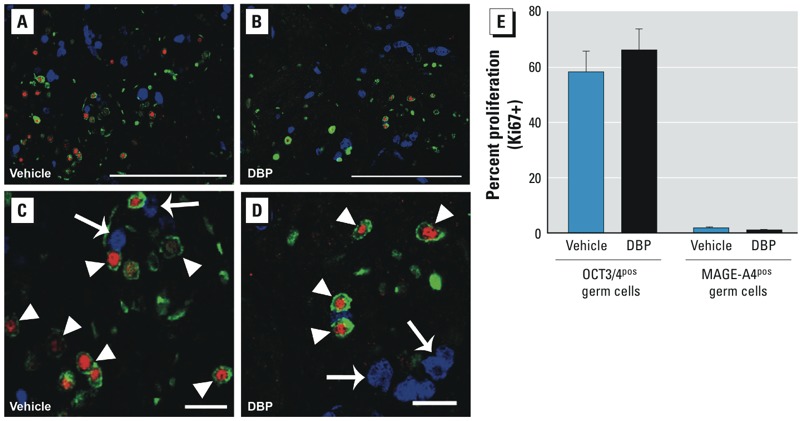
Effects of exposure to vehicle (*A*,*C*) or DBP (500 mg/kg/day; *B*,*D*) on the proliferation (Ki67; green) of undifferentiated (OCT3/4; red) and differentiated (MAGE-A4; blue) germ cell subpopulations in human fetal testis xenografts. (*C*) and (*D*) are higher magnifications of areas in (*A*) and (*B*), respectively. Arrowheads indicate examples of proliferating germ cells expressing OCT3/4; arrows indicate differentiated germ cells positive for MAGE-A4 but negative for OCT3/4 and Ki67 immunostaining. (*E*) Percentage of OCT3/4^pos^ or MAGE‑A4^pos^ germ cells expressing Ki67; no significant differences were found after exposure to DBP. Values shown are mean ± SE for 7 fetuses per treatment. Bars = 200 μm (*A*,*B*) and 50 μm (*C*,*D*).

*Effects of DBP on germ cell proliferation and number in human fetal testis xenografts and on germ cell number in fetal rat testes*. We triple immunostained xenografts for OCT3/4 (undifferentiated germ cell marker), MAGE-A4 (differentiated germ cell marker), and the proliferation marker Ki67 (Figure 6A–D). For vehicle- and DBP-exposed xenografts, the majority of undifferentiated germ cells were immunopositive for Ki67, indicating active proliferation, whereas Ki67^pos^/MAGE-A4^pos^ germ cells were rare (Figure 6E), indicating that germ cells stopped proliferating after differentiation.

**Figure 7:**
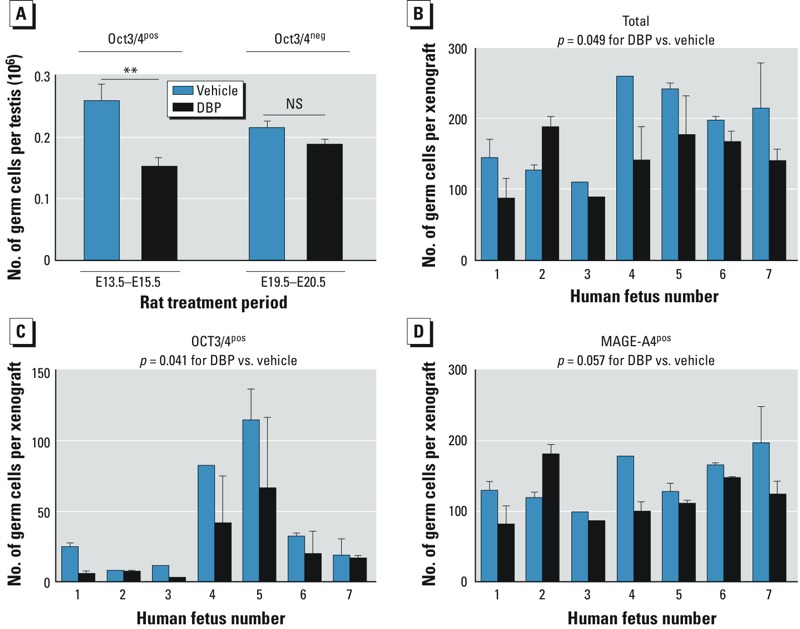
Effect of early (E13.5–E15.5) or late (E19.5–E20.5) fetal DBP exposure (500 mg/kg/day) in rats on germ cell number per testis at E21.5 (*A*). NS, not significant. Effects of exposure to vehicle or DBP (500 mg/kg/day) on the total number of germ cells (*B*) and the numbers of OCT3/4^pos^ (*C*) and MAGE-A4^pos^ (*D*) germ cells in human fetal testis xenografts. In (*B*,*C*,*D*) values shown are the mean ± SE germ cell number for testes from seven individual fetuses (numbered 1–7) xenografted into host mice that were then exposed to vehicle or DBP. Xenograft data were analyzed by two-way ANOVA. **p < 0.01 compared with the respective vehicle group, by unpaired *t*-test.

In rats, exposure to DBP only on E13.5–E15.5 (when germ cells express OCT3/4 and proliferate) reduced the number of germ cells at E21.5, whereas DBP exposure on E19.5–E20.5 (when germ cells have differentiated and stopped proliferating) had no significant effect (Figure 7A). In human fetal testis xenografts, we observed a small but significant reduction in the total number of germ cells per xenograft as a result of DBP exposure (Figure 7B). Similar to the fetal rat, DBP-exposure in human fetal testis xenografts resulted in a significant reduction (*p* = 0.041) in the number of OCT3/4^pos^ germ cells compared with vehicle exposure (Figure 7C), whereas there was a nonsignificant reduction in the number of MAGE-A4^pos^ germ cells (*p* = 0.057; Figure 7D). Thus, DBP exposure resulted in germ cell loss in the human testis during the developmental stage examined (14–20 weeks), with particular loss of undifferentiated OCT3/4^pos^ cells. We found a similar period of sensitivity to DBP-exposure in the rat (i.e., E13.5–E15.5) (Figure 7A).

## Discussion

Concerns about the potential human health effects of phthalates such as DEHP and DBP have been tempered by the demonstration that—unlike the fetal rat testis—the human fetal testis appears to be insensitive to the antisteroidogenic effects of DBP in a range of model systems (Albert and Jégou 2014; Heger et al. 2012; Lambrot et al. 2009; Mitchell et al. 2012; Spade et al. 2014). In contrast, the available evidence suggests that these same phthalates may adversely affect fetal germ cells in both rodents (Ferrara et al. 2006; Jobling et al. 2011) and humans (Heger et al. 2012; Lehraiki et al. 2009; Muczynski et al. 2012), although the underlying mechanisms are unexplored. In the present study, we sought to address these deficiencies by comparing the effects of DBP exposure *in vivo* on germ cells in the fetal rat testis with its effects on germ cells in the human fetal testis in an established xenograft model. Our findings identify periods of germ cell sensitivity to the effects of DBP exposure in the rat (E13.5–E15.5) and human fetal testis (14–20 weeks of gestation). In rats, this coincides with the period when germ cells are undifferentiated (OCT3/4^pos^). In humans, differentiation of germ cells from OCT3/4^pos^ to MAGE-A4^pos^ occurs asynchronously over an extended period from the first trimester to early postnatal life, resulting in a heterogeneous population of germ cells at any given age during fetal development (Mitchell et al. 2008). The predeliction for DBP-induced loss of OCT3/4^pos^ germ cells may indicate a sensitive stage of differentiation rather than gestational age, and this requires further clarification; specifically, there may be more marked effects in earlier gestation when more of the germ cells are undifferentiated. In contrast, the development of germ cell aggregation and/or MNGs was confined to differentiated germ cells in both fetal rats and humans, although aggregation appears to be a rare occurrence in the human compared with the rat. We also observed that the underlying mechanism for DBP-induced germ cell aggregation is probably a loss of Sertoli–germ cell membrane interaction, potentially due to microfilament redistribution within Sertoli cells. These findings provide new insights into the potential human testicular effects of fetal phthalate exposure.

The key finding of our study is that exposure of mice bearing human fetal testis xenografts to a high dose of DBP resulted in a small loss of germ cells, affecting undifferentiated (OCT3/4 expressing) fetal germ cells in particular. Because the undifferentiated, rather than the differentiated, germ cells are highly proliferative (present study; Mitchell et al. 2010, 2014) and because undifferentiated germ cells are present throughout human gestation (Mitchell et al. 2010), it is possible that protracted fetal DBP exposure may cause progressive germ cell depletion in the human fetal testis. Similarly, MEHP exposure *in vitro* causes apoptotic germ cell loss in human fetal testis explants (Lambrot et al. 2009; Muczynski et al. 2012). In our xenograft experiments, we have not investigated the dose dependence of the germ cell changes induced by DBP because of the limited supply of human tissue, but it remains an important issue in terms of risk evaluation for the human fetus. In this regard, we emphasize that in the experiments presented here, we used a dose of DBP (500 mg/kg/day) that is approximately 30,000 times higher than the reported exposure levels for women from the general population (Heudorf et al. 2007). In addition, it is possible that undifferentiated germ cells that survive DBP exposure might be affected in other ways; notably, undifferentiated fetal germ cells are thought to give rise to carcinoma in situ cells in humans, from which testicular germ cell tumors develop in adulthood (Rajpert-De Meyts 2006). The OCT3/4^pos^/MAGE-A4^neg^ population of undifferentiated germ cells has subsequent invasive potential (Mitchell et al. 2014), and this is an important avenue for further study. Overall, our findings show that DBP effects on fetal germ cells in the human are less pronounced than in the rat at the same dose, but the similarity of the types of effect observed with those in the rat suggest that the rat may represent a suitable model for evaluating mechanisms of DBP-induced germ cell effects relevant to the human.

In the fetal rat, induction of MNGs (Fisher et al. 2003; Kleymenova et al. 2005; Mylchreest et al. 2002) and abnormal germ cell aggregation are also consequences of DBP exposure, and as indicated in the present study and by previous studies (Ferrara et al. 2006; Jobling et al. 2011), these effects are confined to differentiated germ cells. We postulate that the formation of MNGs may reflect a failure to maintain cytoplasmic intercellular bridges. Moreover, in the present study, germ cell aggregation was induced by DBP in a dose-dependent manner, even with relatively low doses (20 mg/kg/day). DBP induction of germ cell aggregation was an infrequent occurrence in human fetal testis xenografts, but, as in the rat, it was confined to differentiated (MAGE-A4^pos^) germ cells. Of the seven fetal testes exposed to DBP after xenografting, germ cell aggregates were found in only 4 of 54 xenografts, all of which were derived from a single 19-week fetus. Because undifferentiated and differentiated germ cells are intermixed in the human and there is no defined “early” or “late” period in terms of germ cell differentiation, it may be that induction of aggregation in the human is simply less possible, as opposed to the rodent fetal testes where germ cell differentiation occurs synchronously (McKinnell et al. 2013; Mitchell et al. 2010). Because DBP induction of MNGs in rodents coincides with induction of germ cell aggregation, it may be that the underlying cause is the same. In this respect, the present study as well as previous studies using human fetal testis xenografts (Heger et al. 2012) or *in vitro* cultures (Lehraiki et al. 2009) have shown phthalate induction of MNGs.

The present findings extend previous observations by providing more direct evidence that DBP-induced germ cell aggregation results from dissolution of Sertoli–germ cell membrane cross-linking (via espin–APC), which in turn may result from the redistribution of microfilaments, such as vimentin and N-cadherin. APC, espin, N-cadherin, and vimentin have established roles in Sertoli–germ cell interactions (Bartles et al. 1996; Chen et al. 1999; Kleymenova et al. 2005; Strelkov et al. 2003; Tanwar et al. 2011; Vogl et al. 2008). In the present study, expression of APC and espin was colocalized at the Sertoli–germ cell borders in E17.5 and E21.5 control testes, whereas this colocalization was absent in DBP-exposed testes. Furthermore, immunostaining for the Sertoli cell filament markers N-cadherin and vimentin showed a collapse of the filament structures, with loss of Sertoli cell cytoplasmic “fingers” extending toward the center of the seminiferous cords in DBP-exposed rats at all ages. This altered pattern of vimentin staining following DBP exposure in E21.5 fetal rat testes is similar to that previously described by Kleymenova et al. (2005). Interestingly, the DBP-exposed human fetal testis xenografts that exhibited germ cell aggregation also showed an altered staining pattern for N-cadherin and APC similar to that in the rat, with a lack of Sertoli cell cytoplasmic “fingers” extending around the germ cells and loss of N-cadherin-APC interaction at the membrane level (espin could not be studied in the human). The present findings therefore provide a basis for further studies to investigate the mechanism of DBP-induced germ cell aggregation.

## Conclusions

Our results show that DBP exposure of human fetal testis xenografts results in similar, although more muted, germ cell effects seen in the rat, namely, germ cell loss during fetal life with a predominant loss of undifferentiated (OCT3/4^pos^) germ cells, induction of MNGs, and (infrequent) germ cell aggregation, the latter two effects being confined to differentiated germ cells in both species. Moreover, our results suggest similar DBP-induced loss of Sertoli–germ cell membrane contact among differentiated germ cells. In view of these overall similarities, we suggest that the rat may be an appropriate model in which to study the mechanisms underlying DBP effects on germ cells. Whether fetal germ cell effects would occur in human fetal testes at population-relevant DBP exposure levels remains to be explored.
